# Real-World Use of Clopidogrel and Ticagrelor in Patients With Myocardial Infarction With Nonobstructive Coronary Arteries: Patient Characteristics and Long-Term Outcomes

**DOI:** 10.3389/fcvm.2021.807494

**Published:** 2021-12-21

**Authors:** Side Gao, Haobo Xu, Sizhuang Huang, Jiansong Yuan, Mengyue Yu

**Affiliations:** National Center for Cardiovascular Diseases, Department of Cardiology, Fuwai Hospital, Chinese Academy of Medical Sciences and Peking Union Medical College, Beijing, China

**Keywords:** myocardial infarction with nonobstructive coronary arteries (MINOCA), dual antiplatelet therapy, ticagrelor, clopidogrel, cardiovascular outcomes

## Abstract

**Background:** Current guidelines recommend ticagrelor as the preferred P_2_Y_12_ inhibitor on top of aspirin in patients after an acute coronary syndrome. Yet, the efficacy and safety of ticagrelor vs. clopidogrel in patients with myocardial infarction with nonobstructive coronary arteries (MINOCA) remain uncertain.

**Methods:** A total of 1,091 patients with MINOCA who received dual antiplatelet therapy were enrolled and divided into the clopidogrel (*n* = 878) and ticagrelor (*n* = 213) groups. The primary efficacy endpoint was a composite of major adverse cardiovascular events (MACE), including all-cause death, nonfatal MI, stroke, revascularization, and hospitalization for unstable angina or heart failure. The safety endpoint referred to bleeding events. The Kaplan-Meier, propensity score matching (PSM), and Cox regression analyses were performed.

**Results:** The incidence of MACE was similar for clopidogrel- and ticagrelor-treated patients over the median follow-up of 41.7 months (14.3 vs. 15.0%; *p* = 0.802). The use of ticagrelor was not associated with a reduced risk of MACE compared with clopidogrel after multivariable adjustment in overall (HR = 1.25, 95% CI: 0.84–1.86, *p* = 0.262) and in subgroups of MINOCA patients. Further, there was no significant difference in the risk of bleeding between two groups (HR = 1.67, 95% CI: 0.83–3.36, *p* = 0.149). After PSM, 206 matched pairs were identified, and the differences between clopidogrel and ticagrelor for ischemic endpoints and bleeding events remained nonsignificant (all *p* > 0.05).

**Conclusions:** In this observational analysis of MINOCA patients, ticagrelor was not superior to clopidogrel in reducing ischemic events and did not cause a significant increase in bleeding, indicating a similar efficacy and safety between clopidogrel and ticagrelor. A randomized study of ticagrelor vs. clopidogrel in this specific population is needed.

## Introduction

Dual antiplatelet therapy (DAPT) with aspirin and a P_2_Y_12_ inhibitor remains the cornerstone for secondary prevention in patients after an acute coronary syndrome (ACS) or undergoing percutaneous coronary intervention (PCI) (1–3). Since the publication of the Platelet Inhibition and Patient Outcomes (PLATO) trial and subsequent studies demonstrating the superiority of ticagrelor over clopidogrel in reducing ischemic events without an increase in major bleeding in ACS (4–6), current guidelines have recommended using aspirin with ticagrelor instead of clopidogrel for patients with ACS, unless contraindicated (1–3). Since then, the use of ticagrelor has increased rapidly worldwide. Nonetheless, questions remain about the efficacy of ticagrelor versus (vs.) clopidogrel in different clinical settings. Several randomized trials have found that ticagrelor compared with clopidogrel did not significantly reduce major adverse cardiovascular events (MACE) after fibrinolytic therapy, elective PCI, and among elderly patients with non-ST-elevation ACS (7–9). Recent observational studies also revealed that ticagrelor vs. clopidogrel was not associated with a better prognosis among patients with ACS after PCI in routine clinical practice (10–13). Further, concerns raised about the safety of ticagrelor including the drug-induced dyspnea and higher hemorrhagic risk which may cause early discontinuation, especially among elderly patients and those with more risk factors of bleeding such as anemia and reduced kidney function (14, 15).

As a distinct subpopulation of acute myocardial infarction (AMI), myocardial infarction with nonobstructive coronary arteries (MINOCA) has been increasingly recognized due to the wide use of coronary angiography. Although patients with MINOCA are younger and tend to have fewer comorbidities compared to those with MI and obstructive coronary artery disease (CAD), they are still at considerable risks for long-term cardiovascular (CV) events (16–22). Thus, there is a need to optimize medical therapies in patients with MINOCA, and the antiplatelet strategy is a major part. To date, no relevant study has evaluated the impact of ticagrelor vs. clopidogrel on clinical outcomes after MINOCA. Here, we addressed this issue and compared the efficacy and safety between clopidogrel and ticagrelor in this specific population.

## Methods

### Study Population

**Figure 1:**
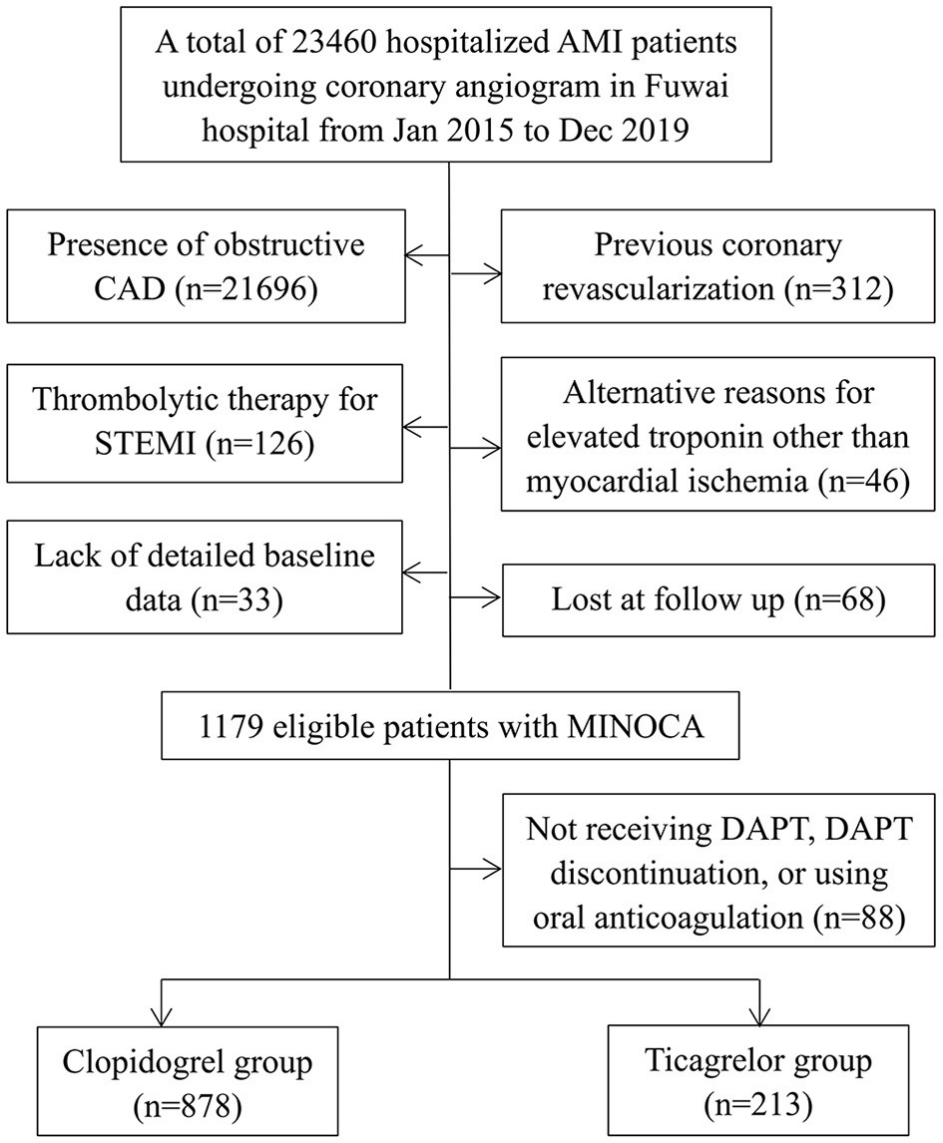
Study flowchart.

This was a single-center, prospective and observational cohort study of patients presenting with MINOCA who received dual antiplatelet therapy (DAPT). A total of 23,460 unique patients with AMI undergoing coronary angiography were consecutively hospitalized in Fuwai hospital from Jan. 2015 to Dec. 2019, including ST-segment elevation myocardial infarction (STEMI) and non-ST-segment elevation myocardial infarction (NSTEMI). MINOCA was diagnosed if patients met the 4^th^ universal definition of AMI (23) and the coronary angiography did not show a stenosis of ≥50% in epicardial coronary arteries (16). Exclusion criteria included: (1) MI with obstructive CAD (*n* = 21,696); (2) prior revascularization (*n* = 312); (3) fibrinolytic therapy for STEMI since coronary artery lesion could be affected by thrombolysis (*n* = 126); (4) alternate explanations for elevated troponin rather than coronary-related myocardial injury (e.g., acute heart failure, myocarditis, takotsubo syndrome, *n* = 46); (5) lack of detailed baseline data (*n* = 33); (6) lost at follow up (*n* = 68); (7) Patients who did not receive DAPT (refused or contraindicated) or discontinued DAPT early and those who needed long-term oral anticoagulation (*n* = 88). As a result, 1,091 eligible MINOCA patients were enrolled into final analysis (Figure 1). All patients were prescribed aspirin (100 mg once daily) and a P_2_Y_12_ inhibitor (clopidogrel 75 mg once daily or ticagrelor 90 mg twice daily) upon admission and for at least 12 months. The P_2_Y_12_ inhibitor was chosen based on the discretion of individual cardiologists. Patients received standard care and the other evidence-based medical treatments, including statins, β-blocker, and angiotensin-converting enzyme inhibitor (ACEI) or angiotensin receptor antagonist (ARB) (3). This study was approved by the Ethics Committee of Fuwai hospital and complied with the Declaration of Helsinki. All enrolled subjects provided the written informed consent.

### Data Collection

Patients' baseline data were collected from medical records. Blood samples were routinely collected from cubital vein under fasting conditions for biochemical test. Serum concentrations of fasting blood glucose (FBG), low density lipoprotein cholesterol (LDL-C), creatinine and high-sensitive C-reactive protein (hs-CRP) were tested by an automatic biochemistry analyzer. The N-terminal pro-B-type natriuretic peptide (NT-proBNP) at admission and peak cardiac troponin I (TnI) values were recorded. Left ventricular ejection fraction (LVEF) was measured by echocardiography using the biplane Simpson method. The Thrombolysis in Myocardial Infarction (TIMI) score was calculated since admission as previously described (24, 25).

### Definitions and Endpoints

In this study, diabetes mellitus (DM) was defined as FBG ≥7.0 mmol/L, 2-h plasma glucose ≥11.1 mmol/L, or having a history of DM. Hypertension was defined as repeated blood pressure ≥140/90 mmHg, past history, or taking anti-hypertensive drugs. Dyslipidemia was diagnosed by medical history or receiving lipid lowering medications.

The primary efficacy endpoint was a composite of major adverse cardiovascular events (MACE), including all-cause death, nonfatal MI, revascularization, nonfatal stroke, and hospitalization for unstable angina (UA) or heart failure (HF). The MACE was assessed as time to first event. The secondary efficacy endpoints included each component of MACE and the composite “hard” endpoint of death, nonfatal MI, stroke, and revascularization. Reinfarction was diagnosed according to the universal definition (23). Revascularization was performed at the operator's discretion due to recurrent ischemia and progression of coronary lesion. Stroke was defined by neurological dysfunction and vascular brain injury caused by cerebral ischemia or hemorrhage (26). Hospitalization for UA or HF reflected the clinical status and quality of life after AMI. The safety endpoints were TIMI bleeding events (27), which include TIMI major and minor bleeding. Patients were regularly followed up at clinics or *via* telephone by independent researchers. All the endpoints were confirmed by at least two professional cardiologists.

### Statistical Analysis

Continuous data were expressed as mean ± standard deviation or median with interquartile range and compared using Student's *t*-test or Mann-Whitney U test. Categorical variables were expressed as numbers with percentages and compared using Pearson's χ^2^ or Fisher's exact test. Cumulative incidence of events were showed by Kaplan-Meier curve and compared using the log-rank test. The Cox proportional regression analyses were performed to identify association between ticagrelor vs. clopidogrel and outcomes. The event risk was adjusted by age and sex in Model 1 and further adjusted by multiple clinically relevant variables in Model 2, including age, sex, MI type (NSTEMI or STEMI), hypertension, diabetes, and dyslipidemia. The hazard ratio (HR) with 95% confidence interval (CI) were calculated. To minimalize the selection bias and control the potential confounding effect of baseline data differences, we used a propensity score matching (PSM) analysis with a one to one match between clopidogrel and ticagrelor groups. Propensity scores were calculated by a binary logistic regression model. We observed that the uneven distribution of baseline risk profiles were mainly due to the differences of age, sex, and MI type, and thus these three factors were enrolled in PSM model. Finally, 206 pairs were identified. The characteristics and outcomes were again compared after PSM. A two-sided analysis with a *P* < 0.05 was considered statistically significant. Data were analyzed using SPSS 23.0 (SPSS Inc.) and STATA 12.0 (StataCorp).

## Results

### Baseline Characteristics

Among MINOCA patients who received DAPT for at least 1 year, 878 received clopidogrel and 213 received ticagrelor (Figure 1). As shown in Table 1, the younger and STEMI patients had a more chance to receive ticagrelor. There were no significant differences in sex, comorbidities, BMI, Killip class, LVEF, TIMI risk score, and the other medications between groups. The FBG, LDL-C, hs-CRP, creatinine, NT-proBNP and TnI values were also similar for both groups. In this regard, the overall risk profiles were similar between clopidogrel and ticagrelor groups.

**Table 1:** Baseline characteristics and clinical outcomes in MINOCA patients treated with clopidogrel or ticagrelor.

**Variables**	**Total (*n* = 1,091)**	**Clopidogrel (*n* = 878)**	**Ticagrelor (*n* = 213)**	***p-*value**
Male, *n* (%)	817 (74.8%)	651 (74.1%)	166 (77.9%)	0.253
Age, years	55.4 ± 11.8	55.7 ± 11.9	53.8 ± 11.1	0.031
BMI, kg/m^2^	25.5 ± 3.8	25.4 ± 3.8	25.7 ± 3.6	0.409
STEMI, *n* (%)	442 (40.5%)	342 (38.9%)	100 (46.9%)	0.033
Past history
Hypertension	580 (53.1%)	470 (53.5%)	110 (51.6%)	0.620
Diabetes	174 (15.9%)	141 (16.0%)	33 (15.4%)	0.840
Dyslipidemia	639 (58.5%)	518 (58.9%)	121 (56.8%)	0.560
Previous MI	58 (5.3%)	43 (4.8%)	15 (7.0%)	0.211
Killip class ≥2, *n* (%)	81 (7.4%)	64 (7.2%)	17 (7.9%)	0.732
LVEF, %	60.5 ± 7.4	60.6 ± 7.6	60.3 ± 6.5	0.606
TIMI risk score	3.4 ± 1.3	3.4 ± 1.4	3.5 ± 1.3	0.124
Blood test
Fasting glucose, mmol/L	5.69 ± 1.69	5.66 ± 1.61	5.82 ± 1.95	0.227
LDL-C, mmol/L	2.30 ± 0.76	2.32 ± 0.77	2.23 ± 0.74	0.165
Creatinine, μmol/L	80.3 ± 18.0	80.8 ± 17.0	82.4 ± 20.3	0.305
hs-CRP, mg/L	2.16 (1.05, 5.84)	2.14 (1.03, 5.38)	2.21 (1.07, 6.73)	0.125
NT-proBNP, pg/ml	376 (115, 692)	371 (107, 683)	382 (121, 715)	0.093
Peak TnI, ng/ml	3.52 (0.93, 6.84)	3.49 (0.88, 6.72)	3.55 (0.96, 7.02)	0.112
In-hospital medication
Statin	1050 (96.2%)	844 (96.1%)	206 (96.7%)	0.687
Beta-blocker	793 (72.6%)	640 (72.8%)	153 (71.8%)	0.755
ACEI or ARB	702 (64.3%)	564 (64.2%)	138 (64.7%)	0.880
CV outcomes
MACE	158 (14.4%)	126 (14.3%)	32 (15.0%)	0.802
Death, nonfatal MI, stroke or revascularization	98 (8.9%)	77 (8.7%)	21 (9.8%)	0.618
All-cause death	16 (1.4%)	14 (1.5%)	2 (0.9%)	0.751
Nonfatal MI	41 (3.7%)	32 (3.6%)	9 (4.2%)	0.689
Revascularization	44 (4.0%)	34 (3.8%)	10 (4.6%)	0.584
Nonfatal stroke	11 (1.0%)	9 (1.0%)	2 (0.9%)	0.910
Hospitalization for UA	65 (5.9%)	54 (6.1%)	11 (5.1%)	0.624
Hospitalization for HF	39 (3.5%)	33 (3.7%)	6 (2.8%)	0.507
Bleeding
TIMI major bleeding	14 (1.2%)	9 (1.0%)	5 (2.3%)	0.124
TIMI minor bleeding	27 (2.4%)	20 (2.2%)	7 (3.2%)	0.395

*BMI, body mass index; STEMI, ST-segment elevation myocardial infarction; LVEF, left ventricular ejection fraction; TIMI, Thrombolysis in Myocardial Infarction; LDL-C, low-density lipoprotein cholesterol; hs-CRP, high-sensitive C-reactive protein; NT-proBNP, N-terminal pro-B-type natriuretic peptide; TnI, Troponin I; ACEI, angiotensin-converting enzyme inhibitor; ARB, angiotensin receptor antagonist; MACE, major adverse cardiovascular events; UA, unstable angina; HF, heart failure*.

### Clinical Outcomes

**Figure 2:**
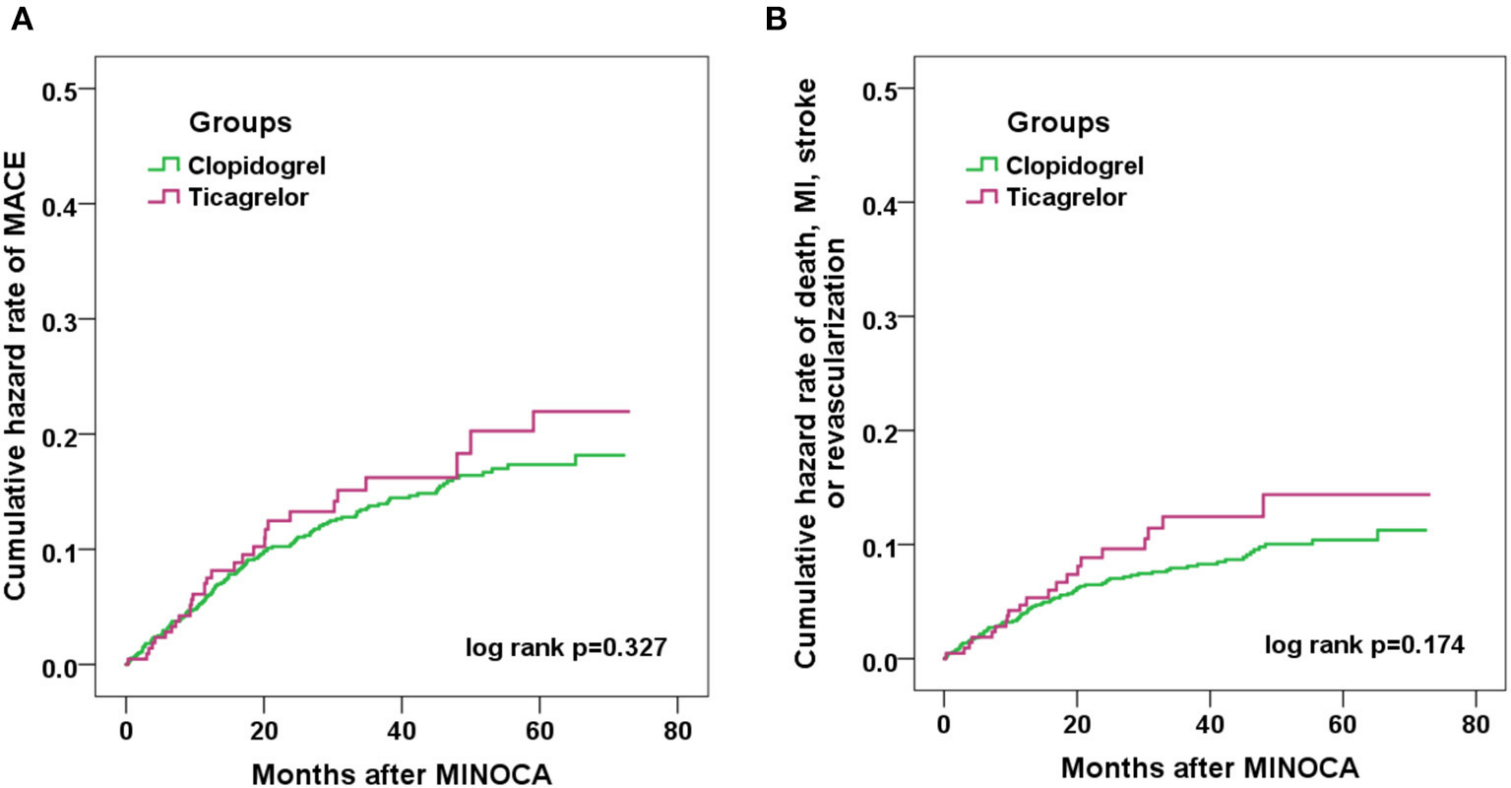
Kaplan-Meier curve analyses showing the cumulative incidence of MACE **(A)** and the composite endpoint of death, nonfatal MI, stroke or revascularization **(B)** in MINOCA patients treated with clopidogrel or ticagrelor.

Over the median follow-up of 41.7 months, 158 patients developed MACE (16 died, 41 had reinfarction, 44 had revascularization, 11 suffered stroke, 65 was hospitalized for UA and 39 for HF) (Table 1). Patients in clopidogrel group had a similar incidence of MACE compared to those in ticagrelor group (14.3 vs. 15.0%; *p* = 0.802). The rate of each individual component of MACE and the composite hard endpoint did not differ significantly between two groups (all *p* > 0.05). The Kaplan-Meier curves (Figure 2) also showed a similar prognosis for both groups (log rank *p* = 0.327 and 0.174 for MACE and the composite hard endpoint). As for safety endpoint, no significant differences in TIMI major or minor bleeding events were observed. After PSM, the demographics and risk factors became comparable among the 206 matched pairs (Table 2). There were no significant differences in the incidence of MACE, the composite hard endpoint, and TIMI bleeding events between clopidogrel and ticagrelor groups after PSM.

**Table 2:** Distribution of clinically relevant variables and adverse events before and after propensity score matching in patients treated with clopidogrel or ticagrelor.

**Variables**	**Pre-PSM**			**Post-PSM**		
	**Clopidogrel (*n* = 878)**	**Ticagrelor (*n* = 213)**	***p*-value**	**Clopidogrel (*n* = 206)**	**Ticagrelor (*n* = 206)**	***p*-value**
Baseline data
Male, *n* (%)	651 (74.1%)	166 (77.9%)	0.253	161 (78.1%)	162 (78.6%)	0.905
Age, years	55.7 ± 11.9	53.8 ± 11.1	0.031	53.8 ± 11.3	53.7 ± 11.0	0.884
STEMI, *n* (%)	342 (38.9%)	100 (46.9%)	0.033	92 (44.6%)	94 (45.6%)	0.843
Hypertension, *n* (%)	470 (53.5%)	110 (51.6%)	0.620	107 (51.9%)	105 (50.9%)	0.844
Diabetes, *n* (%)	141 (16.0%)	33 (15.4%)	0.840	29 (14.0%)	32 (15.5%)	0.677
Dyslipidemia, *n* (%)	518 (58.9%)	121 (56.8%)	0.560	128 (62.1%)	116 (56.3%)	0.229
Previous MI, *n* (%)	43 (4.8%)	15 (7.0%)	0.211	9 (4.3%)	14 (6.7%)	0.283
LVEF, %	60.6 ± 7.6	60.3 ± 6.5	0.606	60.6 ± 6.5	60.3 ± 6.1	0.699
hs-CRP, mg/L	2.14 (1.03, 5.38)	2.21 (1.07, 6.73)	0.125	2.27 (1.16, 5.81)	2.24 (1.06, 6.85)	0.885
NT-proBNP, pg/ml	371 (107, 683)	382 (121, 715)	0.093	369 (103, 688)	378 (114, 702)	0.145
Peak TnI, ng/ml	3.49 (0.88, 6.72)	3.55 (0.96, 7.02)	0.112	3.51 (0.90, 6.83)	3.53 (0.93, 6.94)	0.822
Outcomes
MACE	126 (14.3%)	32 (15.0%)	0.802	30 (14.5%)	29 (14.0%)	0.887
Death, MI, stroke or revascularization	77 (8.7%)	21 (9.8%)	0.618	16 (7.7%)	20 (9.7%)	0.485
Bleeding event	29 (3.3%)	12 (5.6%)	0.109	9 (4.3%)	11 (5.3%)	0.647

*Clinically relevant variables and outcomes were compared before and after propensity score matching (PSM). Baseline risk profiles were comparable after PSM. STEMI, ST-segment elevation myocardial infarction; LVEF, left ventricular ejection fraction; hs-CRP, high-sensitive C-reactive protein; NT-proBNP, N-terminal pro-B-type natriuretic peptide; TnI, Troponin I; MACE, major adverse cardiovascular events*.

### Association Between Treatment With Clopidogrel or Ticagrelor and Outcomes

**Figure 3:**
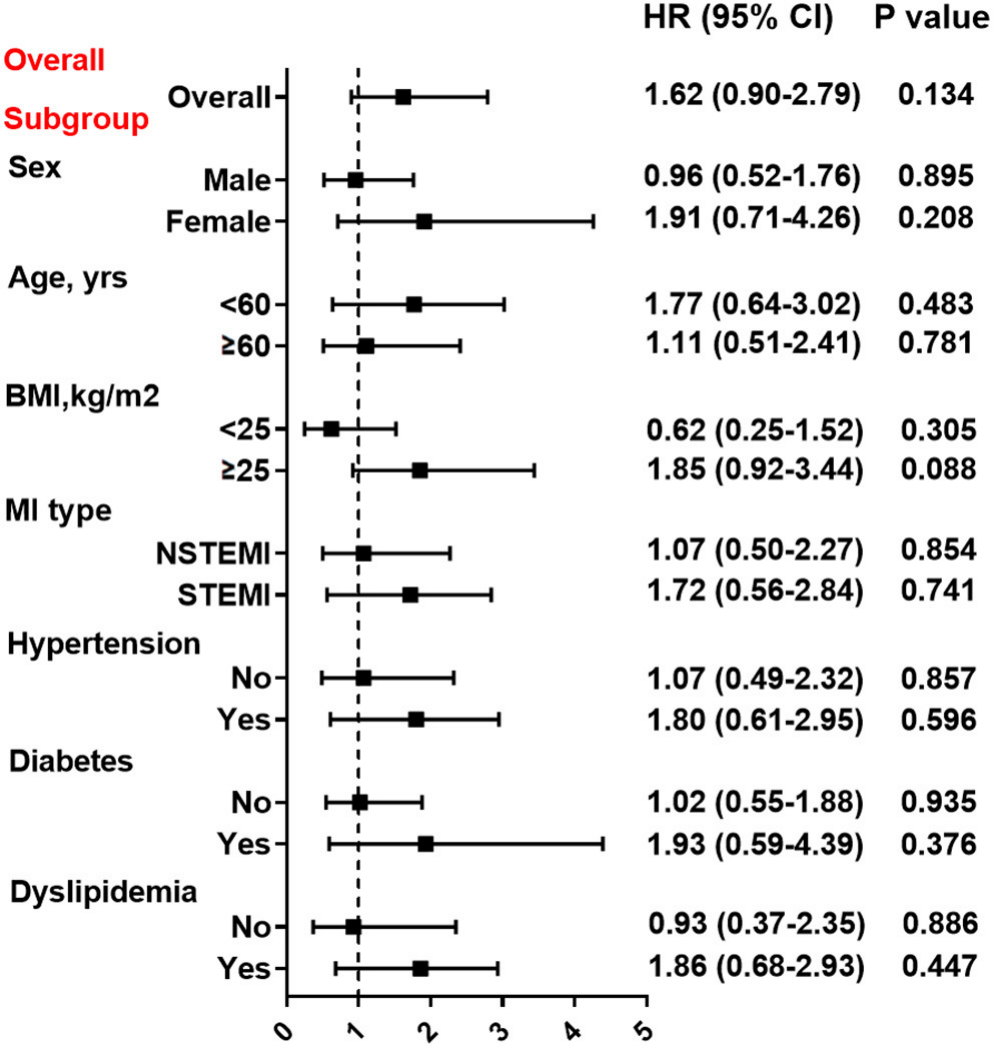
Association between treatment with clopidogrel vs. ticagrelor and MACE risk in overall and subgroups. Subgroup analysis for effect of ticagrelor vs. clopidogrel on MACE risk in patients stratified by sex, age, BMI, MI type, hypertension, diabetes, and dyslipidemia. Hazard ratio (HR) was calculated by the univariate Cox regression analysis. Vertical dotted line indicated the HR value of 1. BMI, body mass index; STEMI, ST-segment elevation myocardial infarction; NSTEMI, non-ST-segment elevation myocardial infarction.

At Cox regression analysis (Table 3), the unadjusted and adjusted risk of events (all *p* > 0.05) before or after PSM were all nonsignificant between two groups. Compared with clopidogrel, the use of ticagrelor was not associated with a reduced risk of MACE (HR = 1.25, 95% CI: 0.84–1.86, *p* = 0.262) or the composite hard endpoint (HR=1.47, 95% CI: 0.91–2.37, *p* = 0.110) even after multivariable adjustment. Furthermore, the risk of MACE for clopidogrel and ticagrelor were similar in a variety of subgroups stratified by the sex, age, BMI, MI type, hypertension, diabetes and dyslipidemia (all *p* > 0.05) (Figure 3). The risk of bleeding events also did not differ significantly between two groups (HR = 1.67, 95% CI: 0.83–3.36, *p* = 0.149). After PSM, still no differences in efficacy or safety endpoints were found between clopidogrel and ticagrelor.

**Table 3:** Impact of clopidogrel vs. ticagrelor on the event risk at Cox analysis.

**Event risk (Tica. vs. Clop.)**	**Pre-PSM**		**Post-PSM**	
	**HR (95% CI)**	***P-*value**	**HR (95% CI)**	***P-*value**
MACE
Unadjusted	1.62 (0.90–2.79)	0.134	1.35 (0.90–2.00)	0.138
Adjusted model 1	1.41 (0.81–2.44)	0.215	1.28 (0.86–1.90)	0.214
Adjusted model 2	1.25 (0.84–1.86)	0.262	1.22 (0.81–1.82)	0.328
Death, MI, stroke or revascularization
Unadjusted	1.54 (0.95–2.49)	0.077	1.32 (0.66–2.64)	0.427
Adjusted model 1	1.50 (0.93–2.44)	0.094	1.27 (0.64–2.54)	0.486
Adjusted model 2	1.47 (0.91–2.37)	0.110	1.19 (0.58–2.44)	0.618
Bleeding
Unadjusted	1.74 (0.87–3.48)	0.113	1.23 (0.50–3.04)	0.647
Adjusted model 1	1.71 (0.85–3.44)	0.132	1.20 (0.48–2.97)	0.686
Adjusted model 2	1.67 (0.83–3.36)	0.149	1.12 (0.44–2.83)	0.804

*Effects of ticagrelor on event risk as compared with clopidogrel before and after PSM were assessed by Cox analysis expressed as HR (95% CI). Model 1 included age and sex. Model 2 included age, sex, MI type (NSTEM or STEMI), hypertension, diabetes, and dyslipidemia in multivariable Cox analysis. PSM, propensity score matching; HR, hazard ratio; CI, confidence interval; MACE, major adverse cardiovascular events*.

## Discussion

In the present study, we investigated the association of ticagrelor vs. clopidogrel with adverse clinical events after MINOCA in a real-world setting, and found that there were no significant differences in MACE or bleeding events between clopidogrel and ticagrelor. These associations remain nonsignificant after PSM, multivariable adjustment, and subgroup analyses, indicating an equivalent efficacy and safety for clopidogrel vs. ticagrelor in MINOCA patients. These data may shed light on the antiplatelet strategies in the contemporary management of MINOCA.

MINOCA represents a distinct clinical entity with multiple pathophysiological mechanisms, including plaque rupture, erosion, thromboembolism, coronary spasm, spontaneous dissection, microvascular dysfunction and supply/demand mismatch. Some non-ischemic diseases such as myocarditis may also mimic the presentation of MINOCA (16). In line with updated guidelines (17, 18), we focused on those with coronary-related ischemia and established a genuine cohort of MINOCA with a long-term follow-up. MINOCA accounts for 5–10% in all AMIs (18), which is close to the prevalence of 5.1% in our study. As reported, nearly one-third of MINOCA would present with STEMI, and patients with MINOCA were more likely to be younger, female, and had fewer comorbidities compared to those with MI-CAD (16). We described the risk profiles of MINOCA as well. Further, we found that the clinical course of MINOCA was not necessarily benign. In our cohort, 1.4% of patients died and 14.4% of them developed a MACE. Previous studies also showed that patients with MINOCA were still at high risk for long-term mortality and CV events despite the optimal medical therapies (19–22). Hence, it is necessary to optimize medical therapies and further improve healthcare for this population.

Antithrombotic treatment is mandatory for ACS patients and those undergoing myocardial revascularization. DAPT consisting of aspirin and a P_2_Y_12_ inhibitor is no doubt the cornerstone. Compared with clopidogrel, ticagrelor is an oral, reversible, direct-acting P_2_Y_12_ inhibitor which has a faster onset of action and exhibits more profound platelet inhibition (1). For decades, the comparative effectiveness of clopidogrel vs. ticagrelor has been addressed by numerous studies, of which the PLATO trial is a landmark research confirming the superiority of ticagrelor over clopidogrel in ACS (4). Based on this convincing evidence, current guidelines have recommend using aspirin with ticagrelor in preference to clopidogrel after an ACS (1–3). Questions remain, however, about the net benefit of ticagrelor compared with clopidogrel in different settings and in real-world clinical practice. Recent randomized or nonrandomized studies further addressed this issue. The TREAT trial showed that ticagrelor did not significantly reduce CV events when compared with clopidogrel in STEMI patients treated with fibrinolysis (7). The ALPHEUS trial revealed that ticagrelor was not superior to clopidogrel in reducing periprocedural myocardial necrosis after elective PCI and did not increase major bleeding (8). The POPular AGE research found that ticagrelor led to more bleeding events without superior net benefit than clopidogrel in elderly Dutch patients (9). In observational studies, some have reported a lower risk of MACE in the ticagrelor group (28–30), while the others did not find a significant difference (10–13). A large Swedish registry showed that ticagrelor use in elderly patients with AMI was associated with higher risk of bleeding and death compared with clopidogrel (10). Further, among patients with ACS who underwent PCI in daily practice, several cohort studies have reported that ticagrelor was not associated with a significant reduction in MACE; instead, it might increase the risk of major bleeding and dyspnea (11–15). These data indicate that the recommendations for ticagrelor in ACS should be applied with caution considering the individual characteristics (e.g., patients treated with elective PCI or thrombolysis, the elderly, and those with higher bleeding risk) and that we may not expect the same efficacy and safety of ticagrelor as evident in clinical trials.

Despite the studies listed above; however, to our knowledge, data regarding the association between use of ticagrelor compared with clopidogrel and clinical outcomes after MINOCA are scarce, and there is an unmet need to optimize antithrombotic strategies in this population. Here, no differences in efficacy or safety were found between clopidogrel and ticagrelor in our cohort. The risk of ischemic or bleeding events between the two groups still did not differ significantly under comprehensive analyses. These findings support the noninferior effect of clopidogrel vs. ticagrelor for net clinical benefit in MINOCA population. Our results are consistent with recent observational studies; yet, they are somewhat in contrast to the PLATO trial. There are several possible explanations. First, the risk profiles were generally comparable among patients treated with ticagrelor or clopidogrel. They had similar clinical conditions, comorbidities and cardiac functions. Second, the benefit *via* stronger platelet inhibition of ticagrelor vs. clopidogrel may be attenuated in MINOCA compared with that in MI-CAD with higher ischemic risk. Previous data showed that patients with diabetes, chronic kidney disease, complex coronary lesions, and high thrombus burden may obtain more benefits from use of ticagrelor (4), whereas the ischemic burden in MINOCA population is not as high as that in the PLATO trial. Third, the overall improvement in clinical outcomes of patients with ACS may also diminish the potential benefit of ticagrelor. For MINOCA patients, this may be particularly driven by advances in healthcare and widespread use of secondary prevention treatments, which may have reduced the need for a stronger P_2_Y_12_ inhibitor. At last, we should note that this is an observational cohort study and we cannot exclude the residual confounding that may have produced this finding. The sample size and the number of adverse events, especially the safety endpoints, are limited. Therefore, our findings should be further validated by a larger randomized study examining the long-term benefit of ticagrelor vs. clopidogrel in MINOCA patients.

## Limitation

Several limitations should be mentioned. First, our cohort was derived from a single-center. The numbers of ischemic and hemorrhagic events may be limited due to the sample size. Thus, future nationwide cohort studies of MINOCA may be more representative. Second, we did not use stringent criteria to select patients that resembles a clinical trial and selection bias may exist. Third, given the observational design of our study, we can only adjust for known risk factors and the residual confounding remains possible although the PSM, multivariate adjustment and subgroup analyses have been performed. Fourth, we did not capture the exact mechanism for every patient. The effect of ticagrelor vs. clopidogrel on outcomes in different phenotypes of MINOCA warrants further research. Fifth, most patients in our cohort would continue the initial P_2_Y_12_ inhibitor prescribed at discharge, still some patients may change their antiplatelet drugs during follow-up. We analyzed the data as intention-to-treat and did not quantify the proportion of patients who switched drugs nor the effects of this. It is possible that some patients crossed over from one drug to another, which may potentially have a bias for the observed associations.

## Conclusion

Among patients with MINOCA receiving DAPT in real-world daily practice, we found that ticagrelor, compared with clopidogrel, was not associated with significant difference in the risk of MACE or bleeding events at a median follow-up of 3.5 years. Future nationwide programs for optimizing antiplatelet strategy in patients with MINOCA are needed and randomized trials are called upon to determine the effectiveness between clopidogrel and ticagrelor in this setting.

## Data Availability Statement

The original contributions presented in the study are included in the article/supplementary material, further inquiries can be directed to the corresponding author/s.

## Ethics Statement

The studies involving human participants were reviewed and approved by the Ethics Committee of Fuwai Hospital. The patients/participants provided their written informed consent to participate in this study.

## Author Contributions

SG conceived and designed the study and drafted the manuscript. SG, HX, and SH performed data analysis and interpretation. JY and MY reviewed and gave final approval of the version to be published. All authors read and approved the final manuscript.

## Funding

This work was supported by National Natural Science Foundation of China (81670415).

## Conflict of Interest

The authors declare that the research was conducted in the absence of any commercial or financial relationships that could be construed as a potential conflict of interest.

## Publisher's Note

All claims expressed in this article are solely those of the authors and do not necessarily represent those of their affiliated organizations, or those of the publisher, the editors and the reviewers. Any product that may be evaluated in this article, or claim that may be made by its manufacturer, is not guaranteed or endorsed by the publisher.
